# Physical Activity and Insulin Resistance in 6,500 NHANES Adults: The Role of Abdominal Obesity

**DOI:** 10.1155/2020/3848256

**Published:** 2020-03-26

**Authors:** James R. Fowler, Larry A. Tucker, Bruce W. Bailey, James D. LeCheminant

**Affiliations:** ^1^Department of Exercise Sciences, Brigham Young University, Provo, Utah 84602, USA; ^2^Department of Nutrition, Dietetics, and Food Science, Brigham Young University, Provo, Utah 84602, USA

## Abstract

This cross-sectional investigation studied differences in insulin resistance across levels of physical activity in 6,500 US adults who were randomly selected as part of the National Health and Nutrition Examination Survey (NHANES). Another important objective was to determine the influence of abdominal obesity on the physical activity and insulin resistance relationship. MET-minutes were utilized to quantify total activity based on participation in 48 different physical activities. Two strategies were employed to categorize levels of physical activity: one was based on relative MET-minutes (quartiles), and the other approach was based on the US physical activity guidelines. Insulin resistance was indexed using the homeostatic model assessment (HOMA). Abdominal obesity was indexed using waist circumference. Effect modification was tested by dividing waist circumferences into sex-specific quartiles and then evaluating the relationship between physical activity and HOMA-IR within each quartile separately. Results showed that relative physical activity level was associated with HOMA-IR after controlling for demographic and demographic and lifestyle covariates (*F* = 11.5, *P* < 0.0001 and *F* = 6.0, *P*=0.0012, respectively). Adjusting for demographic and demographic and lifestyle covariates also resulted in significant relationships between guideline-based activity and HOMA-IR (*F* = 8.0, *P* < 0.0001 and *F* = 4.9, *P*=0.0017, respectively). However, statistically controlling for differences in waist circumference with the other covariates nullified the relationship between total physical activity and HOMA-IR. Effect modification testing showed that when the sample was delimited to adults with abdominal obesity (Quartile 4), relative (*F* = 5.6, *P*=0.0019) and guideline-based physical activity (*F* = 3.7, *P*=0.0098) and HOMA-IR were significantly associated. Physical activity and HOMA-IR were not related within the other three quartiles. In conclusion, it appears that differences in physical activity may play a meaningful role in insulin resistance in those with abdominal obesity, but total activity does not seem to account for differences in insulin resistance among US adults with smaller waists.

## 1. Introduction

Type 2 diabetes is a serious disease. It is associated with increases in an array of comorbidities, including hypertension, depression, coronary heart disease, and obesity [1]. In 2016, 21 million US adults had diagnosed type 2 diabetes, mostly caused by insulin resistance [2]. The 2017 National Diabetes Statistics Report estimated that another 84.1 million US adults had prediabetes in 2015, based on fasting glucose or A1C levels indicative of insulin resistance [3]. Combined estimates of those with diagnosed, undiagnosed, or prediabetes show that these conditions affect an alarming 43.3% of US adults [3].

The disease progression of type 2 diabetes is typically described as the inability of the body to react to the intake of a glycemic load with the correct level of insulin to enable glucose uptake [4, 5]. In most cases, failure of the body to respond with the right amount of insulin occurs because the individual is resistant to insulin [4, 5]. Several factors increase the likelihood of becoming insulin resistant, including obesity, especially abdominal obesity, and physical inactivity [6–8].

Obesity is associated with increased adipose tissue inflammation and changes in circulating concentrations of adipokines, which contribute to insulin resistance in fat, liver, and skeletal muscle tissue [6]. The positive relationship between obesity, insulin resistance, and type 2 diabetes is even more troubling as recent trends show that the prevalence of obesity and severe obesity in US adults has grown from 34% in 2007-2008 to about 40% in 2016. These upward trends further demonstrate the importance of finding effective strategies for the treatment and prevention of insulin resistance [9].

One strategy, which seems to decrease insulin resistance and reduce the risk of type 2 diabetes, is regular physical activity [10]. The decrease seems to occur after chronic exercise, even when the training does not elicit weight loss or a change in body composition [10]. According to some research, when measured objectively, the amount of time engaged in activity is related to increased insulin sensitivity, even in the absence of changes in bodyweight [11].

While it is recognized that physical activity plays a role in reducing the risk of insulin resistance and diabetes, central adiposity also appears to influence these relationships. However, conclusions regarding the nature of the influence of physical activity and abdominal obesity on insulin sensitivity vary [12–16]. Despite the mixed conclusions, evidence shows that the lean and those with little abdominal adiposity experience less insulin resistance and diabetes compared to obese individuals [8,12,17]. These conflicting findings call for further investigation.

With the incidence of obesity steadily increasing and the many negative health consequences that accompany insulin resistance and type 2 diabetes, more research is needed to develop efficacious prevention and treatment strategies. While many studies have found an inverse relationship between physical activity and insulin resistance, the potential mitigating role abdominal obesity plays in this relationship is not clear. Analyzing data from the National Health and Nutrition Examination Survey (NHANES) could help foster a better understanding of the interplay between physical activity, abdominal obesity, and insulin resistance within the US.

The present study had multiple objectives. The first was to identify the relationship between total physical activity and insulin resistance, indexed by HOMA-IR, in a large, nationally representative, sample of nondiabetic adults. Another purpose was to examine the extent to which age, race, sex, smoking, and BMI (body mass index) collectively influence the relationship between total physical activity and insulin sensitivity. The final aim was to examine the extent to which abdominal obesity, indexed by waist circumference, affects the association between physical activity and insulin resistance.

## 2. Methods

### 2.1. Study Design

A cross-sectional study design was employed using data acquired from the National Health and Nutrition Examination Survey (NHANES). NHANES has been a major program of the National Center for Health Statistics (NCHS) since the early 1960s. Data from the hundreds of variables collected by NHANES are used to determine the prevalence of major diseases and risk factors for diseases with the aim of health promotion and disease prevention [18]. NHANES surveys a nationally representative sample of several thousand, noninstitutionalized civilians in the country each year. Collected information is published online as data files and used in a variety of epidemiological studies. Results are helpful in creating public health policies and programs [18]. Data from four consecutive 2-year cycles (NHANES 1999-2000, 2001-2002, 2003-2004, and 2005-2006) were used in this study. After the 2005-2006 cycle, NHANES changed the methods used to measure physical activity; hence, additional years could not be included in the present study. Written consent was obtained from each subject. The Ethics Review Board of the National Center for Health Statistics approved measurement procedures and data collection and posting of the data online for public use.

### 2.2. Subjects

NHANES subjects in the present study ranged from 20 to 84 years of age and had data on participation in 48 physical activities, fasting blood glucose and insulin levels, race, sex, age, year of assessment, body mass index (BMI), smoking status, and waist circumference. Fasting blood glucose and fasting insulin were sample-limiting factors because NHANES required only one-half of the original random sample to fast overnight and provide a fasting blood sample in the morning. A total of 6,500 participants had complete data on each of the variables and were included in the analyses.

### 2.3. Measures

In the current investigation, the exposure variable was MET-minutes of physical activity, indexed using two different methods: relative (quartiles) and guideline-based. Insulin resistance, indexed using HOMA-IR, was the outcome measure. Age, sex, race, year of assessment, BMI, and smoking status were employed as covariates. The key potential confounding variable was waist circumference. Waist circumference was also used to test the presence of effect modification.

#### 2.3.1. Race

The demographic, race, was divided by NHANES into non-Hispanic White, non-Hispanic Black, Mexican American, other races (including multiracial), and other Hispanic categories. Race was used as a covariate in the investigation.

#### 2.3.2. Height

A stadiometer was used to assess the maximum vertical size of participants. With the head free of obstructions, subjects were positioned with head, shoulder blades, buttocks, and heels touching a vertical backboard. Subjects were instructed to look straight ahead, with limbs straight and feet flat on the floor. While the subject took a deep breath and stood as tall as possible, the headboard was lowered, and height was measured. Height measurements were included in the study for the purpose of calculating BMI.

#### 2.3.3. Weight

Overweight and obesity increase the risk of insulin resistance and type 2 diabetes [19,20]. Consequently, body weight data were included in the present study to permit calculation of BMI, which was used as a covariate. Participant weight was measured using a Toledo digital scale. Measurements were taken with subjects wearing minimal clothing, including underwear, disposable paper gowns, and foam slippers [21].

#### 2.3.4. Body Mass Index (BMI)

BMI was used as a covariate in the study. Reductions in BMI are associated with decreased HOMA-IR (homeostatic model assessment of insulin resistance) [22]. BMI allows body weights to be compared, independent of height. BMI was calculated by dividing the subject's weight in kilograms by the square of height in meters [23]. BMI (kg/m^2^) was classified as follows: underweight (BMI: <18.5), normal weight (BMI: 18.5–24.9), overweight (BMI: 25.0–29.9), or obese (BMI: 30.0 and above) [23].

#### 2.3.5. Total Physical Activity

Participants reported the number of days spent participating in each of 48 distinct activities (e.g., aerobics, bicycling, running, hunting, soccer, swimming, tennis, yoga, yard work, and walking) over the past 30 days as well as the typical amount of time spent performing each activity per session, using the NHANES Physical Activity Questionnaire (PAQ) [24]. Self-reported intensity level for each of the 48 activities listed in the PAQ was reported. Intensity levels were defined as either moderate (activities that cause only light sweating or a slight to moderate increase in breathing or heart rate) or vigorous (activities that cause heavy sweating or large increases in breathing or heart rate) [25]. Frequency of participation and time per bout were used to calculate total time spent in each physical activity. Metabolic equivalents (MET-minutes) were then used to index physical activity quantities for each participant. MET-minutes represent the ratio between metabolic rates while engaging in physical activity and at rest [25]. MET-minutes were calculated by determining the MET value of the activity and multiplying by the duration the activity was performed. MET values assigned by NHANES for the specific activities are listed on the NHANES website [24]. Several studies support the validity of the NHANES self-reported physical activity measure, indexed using MET-minutes, since the variable has been shown to be associated with accelerated aging, risk of mortality, and obesity [26–28].

Two methods were used to categorize participant physical activity based on calculated MET-minutes. The “relative method” was based on the distribution of MET-minutes within the large NHANES sample. Participants who reported no physical activity over the past 30 days were classified as Sedentary, comprising 34% of the weighted sample. The remaining participants, who reported some physical activity in the past 30 days, were divided into Low (22.5%), Moderate (22.1%), or High (21.4%) categories as evenly as possible without placing participants with the same MET-minute values in different physical activity categories.

The “guideline-based method” categorized participants into five physical activity categories, according to the 2018 US Physical Activity Guidelines for Americans [29]. Again, 34% of the sample was categorized as Sedentary as they reported no physical activity. Those categorized as Low (21.7%) reported some physical activity but less than 500 MET-minutes per week. Participants with Moderate physical activity (13.7%) reported ≥500 and <1000 MET-minutes per week. Physical activity levels ≥1000 and <1500 MET-minutes per week were categorized as High (9.3%). Participants with Very High physical activity levels (21.3%) reported more than 1500 MET-minutes per week. As with the relative categories, the guideline-based categories were divided as evenly as possible without placing participants with the same calculated MET-minute values in different physical activity categories.

#### 2.3.6. Waist Circumference

Waist circumference was used as a covariate in the study. Waist circumference is strongly associated with insulin resistance [30]. One NHANES study of over 3,500 subjects demonstrated that waist circumference is a significantly better predictor of HOMA-IR, fasting glucose, and HbA1c, than BMI [31]. Waist circumference is an effective and economical way to index abdominal obesity.

Waist circumference measurements were taken with a steel measuring tape extended parallel to the floor, across the iliac crests, and snug without compressing the skin. A wall mirror was used to ensure correct horizontal alignment of the measuring tape and the recorder verified that the measurer positioned the tape parallel to the floor and that the tape was snug. Each waist circumference measurement was recorded to the nearest 0.1 cm [21].

Given the present study was based on a representative sample of 6,500 randomly selected US adults, abdominal obesity was defined as the 75^th^ percentile of the national distribution, as indexed in other NHANES investigations [32]. The 75^th^ percentile is a common threshold employed in epidemiologic research to define elevated risk [33, 34].

#### 2.3.7. Homeostasis Model Assessment (HOMA-IR)

Homeostasis model assessment of insulin resistance is the most common method used to calculate insulin resistance. Increased HOMA-IR has been shown to strongly predict the development of type 2 diabetes, statistically independent of impaired glucose tolerance status, obesity, and body fat distribution [35]. Additionally, higher HOMA-IR has also been shown to be independently associated with the risk of developing prediabetes [36].

HOMA-IR uses the following formula to index insulin resistance: fasting plasma insulin (*μ*U/ml) × fasting plasma glucose (mg/dL)/405. NHANES provided data on participant fasting insulin and fasting glucose measures as well as detailed assessment procedures [37–40].

#### 2.3.8. Smoking

The NHANES smoking file provided data on current cigarette use, history of use, number of cigarettes smoked daily, and other smoking-related details for participants [41]. The cumulative tobacco exposure of NHANES participants was measured in pack years. Packs are comprised of 20 cigarettes each. The number of cigarettes smoked per day was multiplied by the number of total years smoked and then divided by 20 [42]. Pack years of smoking were used as a covariate in this study.

### 2.4. Statistical Analyses

NHANES selects participants using a 4-stage sampling strategy. Therefore, results are generalizable to all noninstitutionalized civilians residing in the United States. In order to generate findings that represent the US population, individual sample weights were applied as part of the analysis process, as recommended by NHANES. Because of the multistage sampling strategy employed by NHANES, instead of thousands of degrees of freedom (df), each of the statistical analyses was based on 59 df in the denominator. The 59 df were derived by subtracting the 58 randomly selected strata from the 117 randomly selected clusters.

Descriptive data were provided by reporting means ± standard errors (SE) for continuous variables and percentages ± SE for categorical variables. Because fasting blood draws were only performed by NHANES on a subsample of those who gave blood, special sample weights were used, as prescribed by NHANES. SurveyMeans was employed with sample weights to calculate means that reflect values for the United States. Similarly, SurveyFreq was utilized to produce prevalence data that reflect values that can be generalized to the United States.

In the present investigation, HOMA-IR was the outcome variable. Individuals who had elevated fasting blood glucose levels, signifying diabetes, were not included in the analyses. Likewise, participants who took medications to control their blood sugar or to influence their insulin sensitivity were not included. The HOMA-IR distribution was found to deviate significantly from a normal distribution; therefore, values were log-transformed.

Total MET-minutes of leisure-time physical activity served as the exposure variable. The relationship between total physical activity and HOMA-IR was determined using linear regression and the SurveyReg procedure. To examine the extent to which the potential confounding variables (i.e., age, race, sex, year of assessment, smoking, BMI, and waist circumference) influenced the physical activity and HOMA-IR association, partial correlation was employed.

Effect modification of waist circumference was tested by dividing waist circumferences into sex-specific quartiles and then evaluating the relationship between total physical activity and HOMA-IR within each sex-specific quartile separately. Partial correlation was also employed within the effect modification evaluation to examine the influence of the potential confounding variables on the relationship between physical activity and HOMA-IR.

Statistical significance was determined using the common 0.05 cut-point, and all *P* values were two-sided. SAS version 9.4 (SAS Institute, Inc., Cary NC) was the computer application employed to generate the statistical outcomes.

## 3. Results

Sample weights provided by NHANES were incorporated into each analysis so that all findings are generalizable to the noninstitutionalized adult population of the United States. The frequencies and weighted percentages for each of the categorical exposure variables and covariates are displayed in Table 1. Participants ranged in age from 20 to 84 with a mean (±SE) age of 44.2 ± 0.4 years. Average (±SE) BMI and waist circumference were 27.8 ± 0.1 kg/m^2^ and 95.5 ± 0.3 cm, respectively. Mean (±SE) physical activity MET-minutes of the sample was 952.1 ± 29.2 minutes per week. Average (±SE) fasting glucose, fasting insulin, and HOMA-IR were 95.1 ± 0.3 mg/dL, 9.3 ± 0.2 mg/dL, and 2.2 ± 0.04, respectively.

According to Table 2, weekly relative physical activity (quartiles) was significantly and inversely related to HOMA-IR after adjusting for age, sex, race, and year of assessment (*F* = 11.5, *P* < 0.0001). After further adjusting for BMI and cigarette smoking, participants in the High-R (High-relative) and Moderate-R categories had significantly lower HOMA-IR than adults in the Low-R and Sedentary-R groups (*F* = 6.0, *P*=0.0012). However, when waist circumference values were controlled simultaneously with the other covariates, there was no relationship between relative physical activity and HOMA-IR (*F* = 1.6, *P*=0.1937).

In Table 3, mean HOMA-IR differed significantly across guideline-based physical activity levels in US men and women with the demographic covariates controlled statistically (*F* = 8.0, *P* < 0.0001). Specifically, adults in the Sedentary-G (sedentary guideline-based) and Low-G physical activity groups differed significantly in HOMA-IR from those in the Moderate-G and High-G physical activity groups, with adults in the Very High-G category differing significantly from all the other physical activity levels. After adjusting for the demographic and lifestyle variables together, the relationship between HOMA-IR and weekly guideline-based physical activity level was weakened, but remained significant (*F* = 4.9, *P*=0.0017). However, after adjusting for all the covariates concurrently, including waist circumference, the relationship was attenuated beyond statistical significance (*F* = 1.7, *P*=0.1673).

To examine the potential modifying effect of waist circumference more comprehensively, the relationship between total MET-minutes of physical activity and insulin resistance was studied across four sex-specific quartiles based on waist circumference. The sex-specific quartiles were labeled small, medium, large, and abdominal obesity, with precisely 25% of the sample in each quartile. The abdominal obesity quartile represented adults ≥75^th^ percentile.

Waist circumference for the quartile labeled small averaged (±SE) 78.1 ± 0.2 cm for men and women combined. Small waist circumferences ranged from 50 cm to 89.25 cm for men and 50 cm to 80.8 cm for women. Men and women in the medium quartile had an average (±SE) waist circumference of 89.5 ± 0.1 cm, with men's waists ranging from 89.25 to 97.6 cm and women's waists ranging from 80.8 to 90.15 cm. The average (±SE) waist circumference for a person in the large waist quartile was 98.9 ± 0.1 cm, with men's waist ranging from 97.6 to 107.25 cm and women's waists ranging from 90.15 to 102.25 cm. Participant waist circumferences in the abdominal obesity quartile averaged (±SE) 115.7 ± 0.4 cm, with men's waists measuring >107.25 cm and women's >102.25 cm, respectively.

Table 4 shows that there were no significant relationships between relative physical activity and HOMA-IR among those with small, medium, or large waists. However, when the sample of 6,500 was delimited to adults with abdominal obesity (Quartile 4), total MET-minutes of relative physical activity and HOMA-IR were related significantly. Specifically, with the demographic covariates controlled, mean HOMA-IR levels between the Sedentary-R and Low-R participants were not different; likewise, there was no difference between the mean HOMA-IR levels of Moderate-R and High-R participants. However, mean HOMA-IR levels differed significantly between the Sedentary-R and Low-R participants compared to the Moderate-R and High-R participants across the relative physical activity categories (*F* = 8.8, *P* < 0.0001). Moreover, after adjusting for the lifestyle covariates, in addition to the demographic variables, the relationship between relative physical activity and HOMA-IR was strengthened between the Sedentary-R and Low-R participants, and the Moderate-R and High-R participants (*F* = 10.5, *P* < 0.0001). Including waist as a covariate with the other covariates weakened the relationship within the sample of individuals with abdominal obesity, but it remained strong and significant.

In Table 5, all the models that focused on guideline-based physical activity and HOMA-IR were significant with the sample delimited to adults with abdominal obesity.

## 4. Discussion

The first objective of the present investigation was to examine the relationship between physical activity, indexed using total MET-minutes per week based on 48 leisure-time activities, and insulin resistance, indexed by HOMA-IR, in a large representative sample of the US adult population. A second aim was to determine the extent to which age, race, sex, year of assessment, smoking, and BMI collectively influenced the relationship between total physical activity and insulin sensitivity. Another key objective was to identify the role waist circumference plays in the association between physical activity and insulin resistance.

Results indicated that after controlling for age, sex, race, and year of assessment, mean HOMA-IR decreased significantly as levels of weekly physical activity increased. Though weakened, this relationship persisted after additionally controlling for BMI and cigarette smoking. However, the relationship between physical activity and HOMA-IR completely disappeared after adjusting for differences in waist circumference simultaneously with the other covariates. These findings suggest that waist circumference mediates the relationship between physical activity level and HOMA-IR. In other words, if all adults had the same waist circumference in the US, physical activity and insulin resistance would not be related. Evidently, insulin resistance decreases as physical activity levels increase in US adults, mostly because active individuals tend to have smaller waists than those who are inactive.

Due to the mediating influence waist circumference seems to have on the relationship between physical activity level and HOMA-IR, the association was examined within each sex-specific quartile of waist circumference. The effect modification findings were enlightening. There was no relationship between physical activity and HOMA-IR among adults with small (Quartile 1), medium (Quartile 2), or large (Quartile 3) waists, considered separately. However, among adults with abdominal obesity (Quartile 4), the association between physical activity and HOMA-IR was strong. Again, differences in waist circumference seem to be a key factor underlying the association between physical activity and insulin resistance.

Physical activity does not seem to play a role in HOMA-IR differences among adults with small, medium, or large waists. However, evidence from the present study suggests that activity level plays a major role in insulin resistance in adults with extra-large waists. In short, although physical activity is important for all adults, when it comes to adults with abdominal obesity, higher amounts of physical activity account for lower levels of insulin resistance.

A 2016 cross-sectional study by García-Hermoso et al. [43] examined the influence of abdominal obesity on the relationship between physical activity and insulin resistance in 1,163 adult men and women randomly selected from outpatient clinics in different regions of Spain. As with the present investigation, García-Hermoso found that controlling for waist circumference completely removed the association between moderate to vigorous physical activity and fasting plasma glucose, fasting plasma insulin, and HOMA-IR [43]. Similarly, a 2006 study by O'Leary et al. [14] concluded that the loss of abdominal visceral fat alone through exercise correlated with decreased insulin resistance. These studies confirm the findings of the present study showing that reduced waist circumference and abdominal adiposity may play a critical role in mediating the relationship between physical activity and insulin resistance [14, 43].

Notwithstanding the studies by García-Hermoso and O'Leary, there is no clear consensus about the mitigating role abdominal fat plays in the relationship between physical activity and insulin resistance. A study by DiPietro et al. [44] found that moderate-intensity aerobic training improved glucose tolerance, independent of changes in abdominal adiposity. Moreover, in a 2007 study on exercise and insulin resistance in obese children, it was determined that exercise alone, independent of body composition changes, reduced insulin resistance [16]. Interestingly, waist circumferences for these children decreased significantly over the exercise training period while DEXA-measured abdominal fat and lean mass remained the same [16].

An overwhelming majority of observational and population-based studies examining the influence of body fat on health have determined that central obesity is the most significant risk factor for insulin resistance and type 2 diabetes (T2D) [45]. The accumulation of excess visceral fat has destructive effects on glucose and insulin metabolism [46]. Obese individuals show increased proliferation of macrophages and increased macrophage participation in inflammatory pathways compared with lean individuals [47]. Visceral fat is a key endocrine organ engaged in the intricate interplay between obesity and systemic inflammation, due partially to its direct hepatic portal access and its ability to secrete greater amounts of proinflammatory adipokines than subcutaneous fat [45, 48]. The literature widely acknowledges that the chronic inflammation associated with obesity induces pancreatic beta-cell dysfunction and insulin resistance [49].

A study by Barzilai et al. [50] demonstrated that visceral fat is a potent modulator of insulin action by surgically removing selective intra-abdominal fat deposits in rats, which improved levels and rates of insulin infusion necessary to maintain plasma glucose levels. Furthermore, a study by Gabriely et al. [51] found that removing visceral fat in rats improved insulin action and delayed the onset of diabetes.

Evidently, not all lipectomies performed in human subjects have shown the link between insulin resistance and visceral fat. One study examining laparotomic gastric bypass with or without omentectomy showed no additional benefit for improved blood glucose levels or serum insulin from the omentectomy surgery [52]. Another similar study found that omentectomy did not enhance the effect of Roux-en-Y surgery on insulin sensitivity, but was associated with preserved insulin secretion, lower circulating C-reactive protein (CRP) levels, and greater weight loss [53]. In a more recent study, significant quantities of mesenteric visceral fat were successfully surgically excised from obese, insulin-resistant baboons, effectively reversing insulin resistance and promoting significant weight loss [45].

In the present study, adults with abdominal obesity (Quartile 4) showed a strong association between higher levels of physical activity and lower HOMA-IR values, while effect modification showed no association between physical activity levels and insulin resistance in adults with small, medium, or large waists, considered separately. It may be that adults with abdominal obesity (i.e., extra-large waists) who participate in regular physical activity are able to decrease the inflammation contributing to increased insulin resistance. Increasing evidence supports the idea that physical inactivity directly causes the inflammation and metabolic dysfunction associated with obesity [54,55]. Furthermore, physical activity is capable of mediating inflammation and metabolic dysfunction without changes in body weight [54]. Moreover, obese individuals typically have two to three times the plasma concentration of inflammatory markers such as interleukin-6 (IL-6) and C-reactive protein of nonobese individuals [54].

Strengths of the present study include its large sample size of 6,500 US adults from the ongoing NHANES study. Participants were randomly selected within the United States. Therefore, the results can be generalized to all civilian, noninstitutionalized adults in the United States. Another strength is that two methods of categorizing physical activity were employed: relative physical activity (quartiles), based on the distribution of MET-minute levels for the NHANES sample, and guideline-based physical activity, founded on the 2018 US Physical Activity Guidelines for Americans [29]. In addition to waist circumference, a number of demographic and lifestyle covariates were controlled, including age, sex, race, year of assessment, BMI, and cigarette smoking. Lastly, evidence of effect modification was tested by examining the activity and HOMA-IR relationship within each sex-specific quartile of waist circumference.

Weaknesses inherent to this investigation include the cross-sectional design of the study, which prohibits the establishment of causal relationships. Additionally, participation in physical activity was assessed using a self-report questionnaire. An objective index produced by pedometers or accelerometers would have likely resulted in a more valid and reliable measure of physical activity. Also, it is possible that participants who reported high levels of physical activity are representative of adults who engage in lifestyles uniquely different from others. Statistical controls were applied to minimize the lifestyle differences, but this risk cannot be eliminated.

## 5. Conclusion

In a random sample of 6,500 US adults, total MET-minutes of physical activity accounted for significant differences in measured insulin resistance. However, the inverse relationship was nullified when participant waist sizes were included in the model, suggesting that the relationship between physical activity and insulin resistance is mediated by abdominal obesity. Moreover, effect modification showed that there was no association between physical activity level and insulin resistance in adults with small, medium, or large waists, considered separately. Nevertheless, the relationship was strong among US adults with abdominal obesity (4^th^ quartile), suggesting that high levels of physical activity may play a meaningful role in glucose and insulin metabolism in those with abdominal obesity (4^th^ quartile), but not in adults with smaller waists.

## Figures and Tables

**Table 1:** Descriptive characteristics of the sample (*n* = 6500).

Variable	*N*	Weighted %	SE
Race			
Non-Hispanic White	3371	73.0	1.5
Non-Hispanic Black	1190	10.4	0.9
Mexican American	1463	7.6	0.8
Other races	213	4.3	0.4
Other Hispanic categories	263	4.7	0.9

Gender			
Men	3090	47.8	0.5
Women	3410	52.2	0.5

Waist circumference			
Small	1447	25.0	0.6
Medium	1555	25.0	0.7
Large	1775	25.0	0.7
Extra-large	1723	25.0	0.7

Body mass index			
Underweight	108	2.1	0.2
Normal weight	2058	34.4	0.8
Overweight	2331	34.2	0.9
Obese	2003	29.4	0.8

Physical activity (relative)			
Sedentary-R	2640	34.0	1.0
Low-R	1354	22.5	0.8
Moderate-R	1309	22.1	0.7
High-R	1197	21.4	0.8

Physical activity (guidelines)			
Sedentary-G	2640	34.0	1.0
Low-G	1326	21.7	0.7
Moderate-G	809	13.7	0.5
High-G	529	9.3	0.5
Very High-G	1196	21.3	0.8

Note: values in the column, weighted %, reflect the distribution of participants after the NHANES sample weights were applied. The physical activity-relative categories were based on the distribution of MET-minute levels for the present NHANES sample. Specifically, participants reporting no regular physical activity were classified as Sedentary, and the remaining adults, each reporting some physical activity in the past 30 days, were divided into sex-specific tertiles. The physical activity-guidelines categories were based on the 2018 US Physical Activity Guidelines. Specifically, Sedentary-G included those reporting no regular physical activity, Low-G included those performing some regular activity, but not reaching the minimum standards of the guidelines, and Moderate-G included those performing ≥500 and <1000 MET-minutes of activity per week, High-G included those performing ≥1000 and <1500 MET-minutes, and Very High-G included those performing ≥1500 MET-minutes of activity per week. Age and smoking (pack-years) were treated as continuous variables in the analyses.

**Table 2:** Differences in mean HOMA values by level of weekly relative physical activity in US men and women, after adjusting for covariates.

Covariate	Weekly relative physical activity level				*F*	*P*
	Sedentary-R	Low-R	Moderate-R	High-R		
	Mean ± SE	Mean ± SE	Mean ± SE	Mean ± SE		
Demographics	2.6^a^ ± 0.08	2.6^a^ ± 0.09	2.2^b^ ± 0.11	2.0^c^ ± 0.08	11.5	<0.0001
Demographics and lifestyle	2.2^a^ ± 0.07	2.2^a^ ± 0.08	1.9^b^ ± 0.09	1.9^b^ ± 0.07	6.0	0.0012
Demographics, lifestyle, and waist circ.	2.7 ± 0.09	2.6 ± 0.08	2.5 ± 0.10	2.5 ± 0.08	1.6	0.1937

^a,b,c^Means on the same row with the same superscript letter were not statistically different (*P* > 0.05). Because of nesting, there were only 59 degrees of freedom in the denominator of each model. The physical activity categories were based on relative MET-minute levels. Participants reporting no regular physical activity were classified as Sedentary, and the remaining adults, each reporting some physical activity in the past 30 days, were divided into sex-specific tertiles. Across the four categories of relative physical activity, weighted percentages were as follows: 34% (*n* = 2640) reported no regular physical activity (Sedentary), 22.5% (*n* = 1354) reported Low levels, 22.1% (*n* = 1309) reported Moderate levels, and 21.4% (*n* = 1197) reported High levels of physical activity (MET-minutes). Because sample weights were applied to each participant, differences in the size of each category should be interpreted relative to percentages, not *N*. Means on the same row were adjusted for the covariates in the left column. Moderate and High mean differences in the demographics model were statistically significant at the *P*=0.0658 level. The demographic covariates were age, sex, race, and year of assessment. The lifestyle covariates were body mass index and cigarette smoking. Waist circ. = waist circumference measured in centimeter.

**Table 3:** Differences in mean HOMA-IR values by level of weekly guideline-based physical activity in US men and women, after adjusting for covariates.

Covariate	Weekly guideline-based physical activity level					*F*	*P*
	Sedentary-G	Low-G	Moderate-G	High-G	Very High-G		
	Mean ± SE	Mean ± SE	Mean ± SE	Mean ± SE	Mean ± SE		
Demographics	2.6^a^ ± 0.08	2.5^a^ ± 0.09	2.3^b^ ± 0.12	2.2^b^ ± 0.16	2.0^c^ ± 0.08	8.0	<0.0001
Demographics and lifestyle	2.2^a^ ± 0.07	2.1^a,c^ ± 0.09	2.0^b,c^ ± 0.09	2.0^a,c^ ± 0.14	1.8^b^ ± 0.07	4.9	0.0017
Demographics, lifestyle, and waist circ.	2.7 ± 0.09	2.6 ± 0.09	2.6 ± 0.11	2.5 ± 0.14	2.5 ± 0.08	1.7	0.1673

^a,b,c^Means on the same row with the same superscript letter were not statistically different (*P* > 0.05). Because of nesting, there were only 59 degrees of freedom in the denominator of each model. The physical activity categories were based on MET-minute guideline levels. Across the five guideline-based categories of physical activity, weighted percentages were as follows: 34% (*N* = 2640) reported no physical activity (Sedentary-G), 21.7% (*N* = 1326) reported Low-G levels (>0 and <500 MET-minutes per week), 13.7% (*N* = 809) reported Moderate-G levels (≥500 and <1000 MET-minutes per week), 9.3% (*N* = 529) reported High-G levels (≥1000 and < 1500 MET-minutes per week), and 21.3% (*N* = 1196) reported Very High-G levels of physical activity (≥1500 MET-minutes per week). Sedentary-G and High-G mean differences in the demographics model were statistically significant at the *P*=0.0667 level. Moderate-G and Very High-G mean differences in the demographics model were statistically significant at the *P*=0.0802 level. Because sample weights were applied to each participant, differences in the number of subjects in each category should be interpreted using percentages, not *N*. Means on the same row were adjusted for the covariates in the left column. The demographic covariates were age, sex, race, and year of assessment. The lifestyle covariates were body mass index and cigarette smoking. Waist circ. = waist circumference measured in centimeter.

**Table 4:** Differences in mean HOMA-IR values by level of weekly relative physical activity in US men and women, after adjusting for covariates applied to waist circumference groups divided into sex-specific quartiles.

Covariate	Weekly relative physical activity level (quartiles)				*F*	*P*
	Sedentary-R	Low-R	Moderate-R	High-R		
	Mean ± SE	Mean ± SE	Mean ± SE	Mean ± SE		
Small waist only						
Demographics	1.2 ± 0.05	1.2 ± 0.06	1.1 ± 0.06	1.2 ± 0.06	0.1	0.9634
Demographics and lifestyle	1.2 ± 0.05	1.2 ± 0.06	1.1 ± 0.06	1.1 ± 0.06	0.3	0.8233
Demographics, lifestyle, and waist circ.	1.2 ± 0.05	1.2 ± 0.06	1.1 ± 0.06	1.2 ± 0.06	0.2	0.9123

Medium waist only						
Demographics	0.4 ± 0.05	0.4 ± 0.05	0.4 ± 0.05	0.4 ± 0.05	0.6	0.6335
Demographics and lifestyle	0.4 ± 0.06	0.5 ± 0.06	0.4 ± 0.07	0.4 ± 0.07	0.3	0.8091
Demographics, lifestyle, and waist circ.	1.8 ± 0.09	1.8 ± 0.09	1.8 ± 0.10	1.8 ± 0.11	0.3	0.8137

Large waist only						
Demographics	2.9 ± 0.17	2.8 ± 0.20	3.0 ± 0.30	2.7 ± 0.20	1.2	0.3241
Demographics and lifestyle	2.8 ± 0.16	2.6 ± 0.19	2.8 ± 0.29	2.5 ± 0.20	2.2	0.0938
Demographics, lifestyle, and waist circ.	2.8 ± 0.16	2.7 ± 0.19	2.9 ± 0.29	2.6 ± 0.20	1.8	0.1639

Abdominal obesity only						
Demographics	4.1^a^ ± 0.24	4.0^a^ ± 0.20	3.1^b^ ± 0.21	3.4^b^ ± 0.25	8.8	<0.0001
Demographics and lifestyle	2.7^a^ ± 0.26	2.6^a^ ± 0.26	1.7^b^ ± 0.28	2.0^b^ ± 0.32	10.5	<0.0001
Demographics, lifestyle, and waist circ.	3.3^a^ ± 0.26	3.2^a,c^ ± 0.24	2.5^b^ ± 0.23	2.7^b,c^ ± 0.27	5.6	0.0019

^a,b,c^Means on the same row with the same superscript letter were not statistically different (*P* > 0.05). Because of nesting, there were only 59 degrees of freedom in the denominator of each model. The physical activity categories were based on relative MET-minute levels. Participants reporting no regular physical activity were classified as Sedentary, and the remaining adults, each reporting some physical activity in the past 30 days, were divided into sex-specific tertiles. Across the four categories of relative physical activity, weighted percentages were as follows: 34% (*n* = 2640) reported no regular physical activity (Sedentary), 22.5% (*n* = 1354) reported Low levels, 22.1% (*n* = 1309) reported Moderate levels, and 21.4% (*n* = 1197) reported High levels of physical activity (MET-minutes). Because sample weights were applied to each participant, differences in the size of each category should be interpreted relative to percentages, not *N*. Means on the same row were adjusted for the covariates in the left column. Low and High mean differences in the demographics, lifestyle, and waist circ. model were statistically significant at the *P*=0.0761 level. The demographic covariates were age, sex, race, and year of assessment. The lifestyle covariates were body mass index and cigarette smoking. Waist circ. = waist circumference measured in centimeter.

**Table 5:** Differences in mean HOMA-IR values by level of weekly guideline-based physical activity in US men and women, after adjusting for covariates applied to waist circumference groups divided into sex-specific quartiles.

Covariate	Weekly guideline-based physical activity level					*F*	*P*
	Sedentary-G	Low-G	Moderate-G	High-G	Very High-G		
	Mean ± SE	Mean ± SE	Mean ± SE	Mean ± SE	Mean ± SE		
Small waist only							
Demographics	1.2 ± 0.05	1.2 ± 0.06	1.1 ± 0.06	1.2 ± 0.07	1.2 ± 0.06	0.9	0.4589
Demographics and lifestyle	1.2 ± 0.05	1.2 ± 0.06	1.1 ± 0.09	1.1 ± 0.08	1.1 ± 0.06	0.9	0.4738
Demographics, lifestyle, and waist circ.	1.2 ± 0.05	1.2 ± 0.06	1.1 ± 0.07	1.2 ± 0.07	1.2 ± 0.06	0.9	0.4985

Medium waist only							
Demographics	1.8 ± 0.06	1.9 ± 0.08	1.9 ± 0.11	1.7 ± 0.08	1.8 ± 0.07	0.6	0.6776
Demographics and lifestyle	1.8 ± 0.10	1.9 ± 0.10	1.9 ± 0.11	1.7 ± 0.11	1.8 ± 0.11	0.4	0.8382
Demographics, lifestyle, and waist circ.	1.8 ± 0.09	1.8 ± 0.10	1.8 ± 0.11	1.7 ± 0.11	1.8 ± 0.11	0.4	0.8435

Large waist only							
Demographics	2.9 ± 0.17	2.8 ± 0.21	2.9 ± 0.27	2.9 ± 0.42	2.7 ± 0.19	0.8	0.5234
Demographics and lifestyle	2.8 ± 0.16	2.7 ± 0.20	2.8 ± 0.26	2.8 ± 0.42	2.6 ± 0.19	1.7	0.1606
Demographics, lifestyle, and waist circ.	2.8 ± 0.16	2.7 ± 0.20	2.8 ± 0.26	2.8 ± 0.42	2.6 ± 0.20	1.3	0.2669

Abdominal obesity							
Demographics	4.1^a^ ± 0.24	3.9^a^ ± 0.21	3.3^b^ ± 0.27	3.3^b^ ± 0.31	3.2^b^ ± 0.26	4.3	0.0039
Demographics and lifestyle	2.7^a^ ± 0.26	2.5^a^ ± 0.27	1.9^b^ ± 0.30	1.9^b^ ± 0.38	1.8^b^ ± 0.33	5.3	0.0011
Demographics, lifestyle, and waist circ.	3.3^a^ ± 0.26	3.1^a^ ± 0.25	2.7^b^ ± 0.24	2.5^b^ ± 0.29	2.6^b^ ± 0.28	3.7	0.0098

^a,b^Means on the same row with the same superscript letter were not statistically different (*P* > 0.05). Because of nesting, there were only 59 degrees of freedom in the denominator of each model. The physical activity categories were based on MET-minute guideline levels. Across the five guideline-based categories of physical activity, weighted percentages were as follows: 34% (*N* = 2640) reported no physical activity (Sedentary-G), 21.7% (*N* = 1326) reported Low-G levels (>0 and <500 MET-minutes per week), 13.7% (*N* = 809) reported Moderate-G levels (≥500 and <1000 MET-minutes per week), 9.3% (*N* = 529) reported High-G levels (≥1000 and <1500 MET-minutes per week), and 21.3% (*N* = 1196) reported Very High-G levels of physical activity (≥1500 MET-minutes per week). Because sample weights were applied to each participant, differences in the number of subjects in each category should be interpreted using percentages, not *N*. Means on the same row were adjusted for the covariates in the left column. The demographic covariates were age, sex, race, and year of assessment. The lifestyle covariates were body mass index and cigarette smoking. Waist circ. = waist circumference measured in centimeter.

## Data Availability

All data used in the present study are available online as part of the National Health and Nutrition Examination Survey (NHANES). The data are free and can be accessed by using the following Centers for Disease Control and Prevention website: https://wwwn.cdc.gov/nchs/nhanes/Default.aspx
